# A Sensor Network Data Compression Algorithm Based on Suboptimal Clustering and Virtual Landmark Routing Within Clusters

**DOI:** 10.3390/s101009084

**Published:** 2010-10-11

**Authors:** Peng Jiang, Shengqiang Li

**Affiliations:** Institute of Information and Control, School of Automation, Hangzhou Dianzi University, Hangzhou 310018, China; E-Mail: lisqou@163.com

**Keywords:** suboptimal clustering, virtual landmark routing within cluster, data compression

## Abstract

A kind of data compression algorithm for sensor networks based on suboptimal clustering and virtual landmark routing within clusters is proposed in this paper. Firstly, temporal redundancy existing in data obtained by the same node in sequential instants can be eliminated. Then sensor networks nodes will be clustered. Virtual node landmarks in clusters can be established based on cluster heads. Routing in clusters can be realized by combining a greedy algorithm and a flooding algorithm. Thirdly, a global structure tree based on cluster heads will be established. During the course of data transmissions from nodes to cluster heads and from cluster heads to sink, the spatial redundancy existing in the data will be eliminated. Only part of the raw data needs to be transmitted from nodes to sink, and all raw data can be recovered in the sink based on a compression code and part of the raw data. Consequently, node energy can be saved, largely because transmission of redundant data can be avoided. As a result the overall performance of the sensor network can obviously be improved.

## 1. Introduction

By integrating different kinds of micro-sensors sensor networks can monitor the environment or designated objects, receive and send information in a wireless way, and transmit real-time information to end users, which makes the idea of information interaction between the real world, computer networks and human society come true [1]. Wireless sensor networks can be applied in areas such as environmental monitoring, intelligent homes, medical care, intelligent transportation, the military, *etc.* The aim of data compression algorithms for wireless sensor networks is to find an efficient way to eliminate redundant data to thus reduce node energy cost and improve the overall performance of the whole system. Meanwhile, the accuracy of recovered data must be assured. Many data compression algorithms for wireless sensor networks have been proposed recently. Jia and Guevara have proposed the so-called Group-Independent Spanning Tree algorithm, which constructs a complete data transmission structure, and aims to reduce the spatial redundancy of data [2], but their discussion of algorithm parameters and redundancy elimination within clusters is insufficient. A self-based regression algorithm proposed by Deligiannakis first splits the recorded series into intervals of variable length [3], then these intervals are encoded based on an artificially constructed base signal, so the base signal and the extent of piece-wise alignment of recorded series are critical factors of the algorithm. Song and Kalogeraki have proposed an online information compression algorithm [4] which first divides data obtained by nodes into different shorter length datasets. Then a dictionary can be established according to the shorter data segments and updated with the increased data obtained by nodes. The algorithm can reduce the average energy cost of the nodes and improve the accuracy of recovered data, however, the need to update the dictionary makes the algorithm more complex than the self-based regression algorithm discussed above. Chou and Petrovic have proposed a distributed structure tree depression algorithm [5], which explores the spatial-temporal correlativity existing in data and computes the correlativity parameters in the sink. Then the sink recovers data according to these correlativity parameters and part of the raw data transmitted from nodes, so the energy cost of nodes can be reduced to some extent. However, the number of raw data groups transmitted from nodes to the sink is not defined when estimating correlativity parameters. If a node transmits too much raw data for estimating correlativity, some redundant parameters will be imported, then node energy will be wasted and accuracy will be affected. On the other hand, if the raw data transmitted for estimating correlativity is not enough, the accuracy of data recovered at the sink will be seriously affected. An algorithm for multidimensional sequential pattern mining over data streams was proposed by Chedy and Plantevit in [6]. It scans the temporal series record and realizes data coding based on pattern frequency. Meanwhile, multidimensional sequential patterns extracted from the data can be used to compress it. Because data need to be scanned, memory must be large enough and energy cost of nodes and the real-time capability of the system will be affected. Zhou and Lin have proposed a distributed spatial-temporal data compression algorithm based on ring topology wavelet transformation [7], which supports a broad scope of wavelet transformations and is able to efficiently decrease spatial-temporal redundancy, but the algorithm may be complex for nodes because of the wavelet transformations. A data compression algorithm based on route selection proposed by Pattem and Krishnamachari combines routing with data compression [8]. The algorithm reduces the data space redundancy by balancing the data correlativity of adjacent nodes and the size of clusters. Then the average energy cost of nodes will be decreased and the longevity of the system will be increased. However, the algorithm needs to set the accurate position of nodes and the distance of different nodes should be same, which is hard to fulfill in practice. Meanwhile, the correlativity of data decides the performance of the algorithm and it is hard to get correlativity parameters in advance of the application. Wang and Hsieh have proposed a multi-resolution spatial and temporal coding algorithm (abbr. MSTC) [9], which first eliminates temporal redundancy by comparing data obtained from nodes with a threshold. Historical data will be stored by means of exponential increasing. That is to say, the smaller the interval to the present moment, the more raw data will be stored in a node. Then the networks will be clustered based on a virtual grid. Spatial redundancy will be eliminated through a discrete cosine transform. However, for the limited node resources, a discrete cosine transform is complex and the energy cost for the node is too large.

Aiming to reduce the temporal-spatial redundancy that exists in data obtained by nodes, this paper proposes a data compression algorithm for wireless sensor networks based on suboptimal clustering and virtual landmark routing within clusters (abbr. SC-LVLR). Suboptimal clustering is used to divide the whole network according to the spatial correlativity, which is the basis for eliminating spatial redundancy. On the other hand, optimal routes are established based on virtual landmarks in the clusters and a structure tree established between the clusters, respectively. During the course of transmitting data from nodes to clusters and from clusters to a sink, redundant data existing in adjacent nodes and adjacent clusters are both eliminated, so the data transmitted from nodes to the sink are greatly reduced and the average energy cost of nodes can obviously be reduced too. Meanwhile, the ability of locating an exceptional node in the whole system can be improved and the longevity of sensor networks can be extended.

The second part of this paper will illustrate suboptimal clustering theory and virtual landmark routing within the cluster model established by SC-LVLR. Because the MSTC algorithm also aims to eliminate data temporal-spatial redundancy and represents a class of temporal-spatial redundancy-eliminating algorithms, we compare SC-LVLR and MSTC, and the corresponding flow charts are given in the third part. In the fourth part, the average energy cost per node, signal to noise ratio and the number of expired nodes are taken as criteria to test the performance of the different algorithms and the algorithm performance is analyzed in detail through simulation examples. Finally, our conclusions and an outlook for the future are given.

## 2. Suboptimal Clustering and Virtual Landmark Routing within Cluster

Dividing the whole sensor network based on the spatial correlativity of data obtained by nodes is not only beneficial for eliminating spatial redundancy but also good for locating exceptions. However, given the diversity of practical applications, sensor networks’ size and number of monitored objects, it is difficult to propose an optimal algorithm that suits all kinds of situations based on the limited energy, memory and computing ability of nodes, so the suboptimal clustering algorithm from reference [10], which is simple for nodes and able to realize suboptimal clustering in a large variety of networks of different sizes through model analysis and simulation tests is introduced. To cluster sensor networks, both the spatial correlativity of data obtained by nodes and the size of the routing table consisting of the cluster heads should be considered simultaneously. If the clusters are too small, the routing table will be enlarged and searching for optimal routing will be more complex for nodes. Conversely, if the cluster is too large, nodes far away from each other have little spatial correlativity, which is not beneficial for eliminating spatial redundancy. Meanwhile, cluster head will need to collect so much data from nodes within a cluster, that the cluster head expire quickly and this will not be beneficial for balancing the energy cost of the whole sensor network.

The suboptimal clustering algorithm supposes the information obtained by nodes can be expressed by *H_i_*. Normally, the combined information entropy corresponding to data obtained by two arbitrary nodes in a sensor network can be expressed by a function of the distance between the two nodes. When the distance of two nodes is *d*, the average united information entropy is given by the following expression:

(1) H2(d)=H1+[1−1(dc+1)]H1

From formula (1), we know that average irrelative information supplied by every new node is [1−1(dc+1)]H1 for an existing information source, where *d* stands for the least distance from the new node to the existing information source and *c* stands for the spatial correlativity parameter of node. The information entropy produced by a set consist of *n* nodes can be estimated through the iteration method. A set of nodes is initialized as *S*_1_ = {*v*_1_}, where *v*_1_ represents an arbitrary node. We can get *H*(*S*_1_) = *H*_1_ when we set the combined united information entropy of node set *S_i_* as *H*(*S_i_*). Suppose *V* as a set includes all nodes, and set *S*_*i*−1_ can be updated by adding node *v_i_* (*v_i_ ∈ V*). Node *v_i_* is chosen on the condition that node *v_i_* is out of set *S*_*i*−1_ and the sum of Euclidean distance from *v_i_* to every node of set *S*_*i*−1_ is the least. Set *S_i_* = (*S*_*i*−1_*, v_i_*) and *d_i_* stands for the least Euclidean distance from node *v_i_* to set *S*_*i*−1_. Namely, *d_i_* formulates the least sum of the Euclidean distances from *v_i_* to every node of set *S*_*i*−1_. Combined entropy can be shown as follows:

(2) H(Si)=H(Si−1)+[1−1(dic+1)]H(S1)

Then we can get *H*(*S_i_*) as the estimation of *H_n_*. When all nodes are deployed evenly at a distance *d* formula (2) can be simplified into formula (3), which is an effective estimation expression for a linear situation:

(3) Hn(d)=H1+(n−1)[1−1(dc+1)]H1

To divide sensor networks effectively, we can begin with a simple situation. Supposing *n* nodes included in a sensor network are deployed evenly on a two-dimensional grid. Every cluster includes *s* nodes and the distance of adjacent nodes is *d. D* stands for the number of hops from cluster head to sink along the shortest route. Because information entropy is related to the data that must be transmitted and the amount of data is connected with the energy cost of nodes, the total energy cost when a cluster includes *s* nodes can be calculated as follows:

(4) $$E_{s}(c)=\frac{n}{s}(E_{\mathrm{in}}+E_{\mathrm{extra}})$$

In formula (4), *c* stands for spatial correlativity parameter. *E_in_* and *E_extra_* stand for the energy costs of transmitting data within and out of a cluster, respectively:

(5) $$E_{\mathrm{in}}=\sum_{i=1}^{s}H_{i}=\sum_{i=1}^{s}\left(1+\frac{i-1}{1+c}\right)H_{1}=(s+\frac{(s-1)s}{2(1+c)})H_{1}$$

(6) $$E_{\mathrm{extra}}=(1+\frac{s-1}{1+c})H_{1}D$$

The derivation of formulas (5) and (6) can be found in [10]. The total energy can be obtained as follows:

(7) $$E_{s}(c)=nH_{1}[1+\frac{\left(s-1\right)}{2(1+c)}+\frac{D}{s}+\frac{(s-1)D}{s(1+c)}]$$

For realizing optimal clustering, we can get formula (8) through differentiation of *E_s_*(*c*) by *s*:

(8) $$s_{\mathrm{optimal}}=\sqrt{2\mathrm{Dc}}$$

From formula (8), we can find that optimal clustering solution *s_optimal_* is dependent on the hops from cluster head to sink and the spatial correlativity of the data. It is difficult to determine the number of hops from cluster head to sink and quantify the spatial correlativity of data in practical applications, so here we search for a suboptimal solution here. Set:

(9) $$E^{*}(c)=E_{S_{\mathrm{optimal}}}(c)$$

We need to minimize the difference of optimal energy cost E*(*c*) and suboptimal energy cost *E_Snop_*(*c*). Namely,

(10) $$\underset{s}{\text{min}}\underset{c\in [0,\infty )}{\text{max}}\left|E_{S_{\mathrm{nop}}}(c)-E^{*}(c)\right|$$

For getting a suboptimal solution, we consider two extreme cases. Namely, *c* formulas 0 and ∞, respectively. Set:

(11) $$E_{S_{\mathrm{nop}}}(0)-E^{*}(0)=E_{S_{\mathrm{nop}}}(\infty )-E^{*}(\infty )$$

According to formula (7), we can get:

(12) $$E_{S_{\mathrm{nop}}}(0)=nH_{1}[1+\frac{\left(s-1\right)}{2}+D]$$

(13) $$E_{S_{\mathrm{nop}}}(\infty )=nH_{1}[1+\frac{D}{s}]$$

in the extreme situations, namely, set *S* = 1, when different nodes have no correlativity, and *S* = *n* when the spatial correlativity is limitless. Then we can get formula (14) and formula (15) as follows:

(14) $$E^{*}(0)=nH_{1}[1+D]$$

(15) $$E^{*}(\infty )=nH_{1}[1+\frac{D}{n}]$$

According to formulas (11) to (15), we can get:

(16) $$\left(1+\frac{\left(s-1\right)}{2}+D-(1+D))=(1+\frac{D}{s}-(1+\frac{D}{n})\right)$$

Namely:

(17) $$\frac{\left(s-1\right)}{2}=\frac{D}{s}-\frac{D}{n}$$

Then for simplifying computation, we set *D* = *n* in formula (17), and we can get the suboptimal solution as follows:

(18) $$s_{\mathrm{nop}}=\frac{\sqrt{8n+1}-1}{2}$$

The basic theory about virtual landmark routing within clusters will be demonstrated in the following section. The routing method is based on the connectivity of nodes in clusters and the virtual landmarks of cluster heads to realize routing in clusters, thus avoiding the dependence on practical landmarks of nodes. Meanwhile, a global structure tree rooted from the sink is built among cluster heads and a global routing table can be obtained. Supposing *G* = (*V*,*E*) is a communication network consisting of some nodes. *V* stands for the set of all nodes and *E* stands for the set of un-weighed communication lines. *χ*(*p,q*) stands for the least routing distance (namely the number of routing hops) of arbitrary nodes *p* and *q* in set *V*. *s*⊂*V* is a subset of the set cluster heads, and the Voronoi set *δ*(*p*) of arbitrary node *p* in set *V* can be defined as follows:

(19) δ(p)={q∈V|∀u∈S,χ(q,p)≤χ(q,u)}

*Theorem 1*. For an arbitrary node *q* in a Voronoi set *δ*(*p*), the least distance between nodes *p* and *q* is included in the set *χ*(*p,q*).

This theorem can be proved by contradiction and the details can be found in reference [11]. For a given set of cluster heads, the Voronoi set of every cluster head includes some of the network nodes. If cluster heads are chosen properly, every Voronoi set will be connected and the topology routing from nodes to cluster head is relatively simple, so the routing of complex sensor networks can be divided into a simple global routing problem based on cluster heads and local routing in the clusters. The ordinary network routing strategy is to set a global coordinate for every node and then execute a greedy algorithm based on Euclidean distance to realize the transmission of data. However, with their limited energy and computing ability, it is difficult for nodes to get and store all nodes’ global coordinates, so the notion of a virtual landmark within clusters is introduced to solve the above problem in situations of continuation and discreteness, respectively.

In the case of continuation, the distance between nodes is expressed by the Euclidean distance. The aim of setting virtual landmarks within clusters is to build a coordinate function set, which is only dependent on the Euclidean distances of different cluster heads. Along the direction of gradient descent of the coordinate function set, the target node is always reachable. Supposing {*u_i_*} is a set consisting of *n* cluster heads in the same plane; then the virtual coordinate vector of an arbitrary node *k* in the same plane can be expressed as follows:

(20) $$V_{a}(k)=(\left|k-u_{1}\right|,\left|k-u_{2}\right|,\cdots ,\left|k-u_{n}\right|)$$

In formula (20), |k- *u_i_*| stands for the Euclidean distance from *k* to *u_i_*. The distance of *q* and *p* in the virtual coordinate systems can be expressed as follows:

(21) $$d(p,q)={\left|V_{a}(p)-V_{a}(q)\right|}^{2}={\sum_{i=1}^{n}\left(\left|p-u_{i}\right|-\left|q-u_{i}\right|\right)}^{2}$$

However, under the condition of *n*>9, along the direction of gradient descent of *d*(*p,q*) of target node *p*, the routing may get trapped in a local loop when executing the greedy routing algorithm, so an adjustment is needed for formula (20). We set:

(22) $$V_{b}(k)=({\left|k-u_{1}\right|}^{2},{\left|k-u_{2}\right|}^{2},\cdots ,{\left|k-u_{n}\right|}^{2})$$

The virtual landmark vector of arbitrary node *k* can be adjusted by formula (23). *V̅_b_*(*k*) is the average of *V_b_*(*k*).

(23) $$V(k)=\left\{{\left[V_{b}(k)\right]}_{1}-{\bar{V}}_{b}(k),\;{\left[V_{b}(k)\right]}_{2}-{\bar{V}}_{b}(k),\;\cdots ,{\left[V_{b}(k)\right]}_{i}-{\bar{V}}_{b}(k)\right\}$$

Under the centering virtual coordinate system, the distance of *q* and *p* can be expressed as follows:

(24) $$d(p,q)={\left|V(p)-V(q)\right|}^{2}$$

*Theorem 2*. If at least three cluster heads which are non-collinear exist on the continuous Euclidean plane, along the direction of gradient descent of *d*(*p,q*) the target node *q* is always reachable. The proof of Theorem 2 can be found in reference [11]. Euclidean distance discussed above is extended to hops, in the case of discreteness. Suppose a set of cluster heads as {*u_i_,i* = 1, ⋯ *n*} and *φ*(*k,u_i_*) as the least number of hops from *k* to *u_i_* for an arbitrary node *k.* Set:

(25) $$\bar{\phi (k)}=\sum_{i=1}^{n}\phi {\left(k,u_{i}\right)}^{2}/n$$

(26) $$V^{\prime }(k)=\left\{\phi {\left(k,u_{1}\right)}^{2}-\bar{\phi (k)},\phi {\left(k,u_{2}\right)}^{2}-\bar{\phi (k)},\cdots ,\phi {\left(k,u_{n}\right)}^{2}-\bar{\phi (k)}\right\}$$

Under the centering virtual coordinate system, the distance of nodes *p* and *q* can be expressed as:

(27) $$d(p,q)={\left|V^{\prime }(p)-V^{\prime }(q)\right|}^{2}$$

Up to now, the local virtual coordinate system is built and the routing from node to sink can be divided into two parts, namely, within and outside cluster routing. Compared with the number of nodes, the number of cluster heads is relative small, so a global network structure tree rooted from the sink can be built. Then the least hops routing table will be broadcasted to all cluster heads. Routing in clusters includes non-edge region and edge region routing. Non-edge region routing can be obtained along the direction of gradient descent according to the virtual landmark within a cluster. The choice of edge region routing sets the neighbor cluster head which is in the direction of gradient descent as the target node before data is transmitted across the cluster border and executes non-edge region routing after the data is transmitted across the cluster border.

## 3. Algorithm Description and Flow Chart

**Figure 1.**
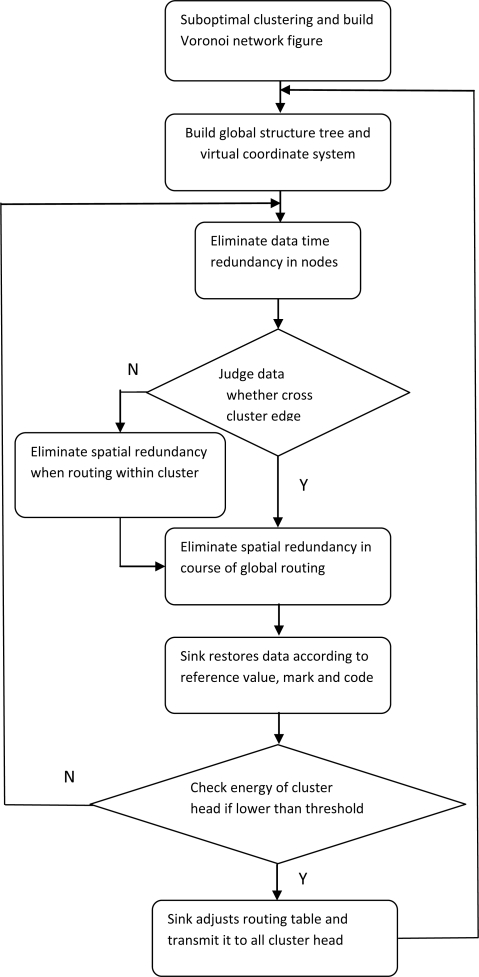
Flow chart of the SC-LVLR algorithm.

**Figure 2.**
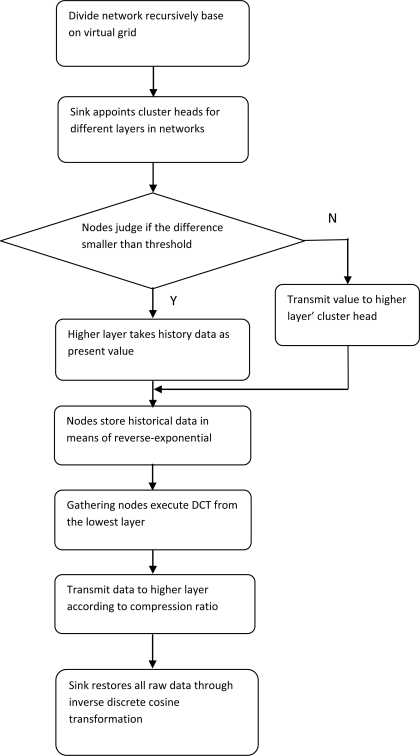
Flow chart of the MSTC algorithm.

Based on the theories of suboptimal clustering and virtual landmark routing within clusters clarified above, details of the algorithm proposed in this paper will be demonstrated in the following section. Firstly, the difference of monitoring values obtained by the same node in sequential moments will be coded to eliminate the time redundancy. Then spatial redundancy will be eliminated in courses of data transmitted from nodes to cluster heads and from cluster heads to sink in the same way. The Multi-resolution Spatial and Temporal Coding (MSTC) algorithm proposed by Wang and Hsieh realizes data compression by eliminating temporal-spatial redundancy, and stands for a class data compression algorithm in wireless sensor networks. It will be illustrated necessarily here for comparison with the algorithm proposed in this paper. Flow charts of SC-LVLR and MSTC are shown in Figure 1 and Figure 2, respectively. Concrete steps for realizing SC-LVLR are as follows:

1. Sensor networks are divided into some clusters based on suboptimal clustering theory, and cluster heads are randomly appointed by the sink at first. A Voronoi network is established and then edge region nodes will be formed.
2. A structure tree rooted from the sink and including all cluster heads will be established. Meanwhile, a virtual coordinate system is built within clusters on the condition that the cluster is appointed as reference node. Cluster head and nodes within the cluster are imparted a global mark and a local mark, respectively.
3. Time redundancy will be eliminated through coding the difference between the present instant monitor value and values stored in some pre-instance (the amount of data stored depends on the actual application and the memory of the node). This way a group of raw monitoring values can be replaced by reference values and compression code.
4. Routing in clusters can be realized through a greedy algorithm based on a virtual coordinate system. Spatial redundancy can be eliminated in a similar way by eliminating time redundancy in the course of routing within clusters. The difference is that the next hop node is set as reference node. The reference node local mark will be included and only time redundancy need be compressed around monitoring values obtained by the cluster head.
5. If the cluster heads have gathered all compressed data within a cluster, then compressed data will be transmitted from the cluster head to a sink along the route in the global table. In the course of transmitting data, if data have reached edge region nodes, edge region routing discussed above will be executed, or non-edge region routing will be executed. Spatial redundancy existing in adjacent clusters will be eliminated when data cross the edge region. As a reference, clusters which are nearby the sink won’t execute the spatial redundancy compression. As a supplement in case that routing within cluster is trapped into a local loop, flooding routing will be executed.
6. According to reference values, compression code and the global marks transmitted from cluster heads, nodes where spatial compression code is produced can be ascertained. The sink first decodes the spatial compression code according to the mapping relationship between code and difference, and then decodes the time compression code. In combination with reference values, raw data can be recovered in the sink.
7. The sink will check the energy of cluster heads periodically. If the remaining energy is lower than a threshold, the node with most energy in the cluster will be elected to replace the raw cluster head, and the global routing table will be regulated by the sink and broadcast to all cluster heads, and then step (2) or step (3) will be executed.

For comparison, the realization steps of MSTC will be as follows:

1. The sensor networks are divided into k*k (the value of k depends on the practical application) parts evenly based on a virtual grid. Every part will be divided into k*k parts recursively until the smallest virtual grid only includes one node. Since the deployment of nodes is random, the smallest virtual grid may include zero, one or more nodes. If more than one node is included in the same smallest virtual grid, average value will be regarded as the monitoring value of the smallest virtual grid. If there is no node included, an interpolated value from an adjacent grid will be regarded as the grid value.
2. The smallest virtual grid is in the lowest layer, and cluster head will be elected among the k*k adjacent smallest virtual grids. Recursively, different cluster heads in different layers will be elected. The layer structure tree is established in the whole network.
3. If the interval is set as T and the value obtained in the moment p needs to be transmitted to cluster heads in a higher layer, then values obtained at moments p + n*Ṭn = 1,2,3…) will need to be transmitted to cluster heads in higher layer. Values obtained between the moments p and p + T will be compared with the value obtained at moment p; if the difference is smaller than a threshold, values will be considered the same as historic values in the higher layer and there is no need to transmit data to the higher layer, or if different they will be transmitted to the higher layer.
4. Since the memory of nodes is limited, historic values are stored by reverse-exponential means, namely, values obtained at moments t-1, t-2, t-4, t-8 and t-16 will be stored if the present moment is t. That is to say, the nearer values obtained are from the present moment, the higher the possibility the value will be stored in nodes.
5. To eliminate spatial redundancy, starting from the lowest layer, k*k adjacent virtual grids will be mapped into a k*k matrix, which will be processed with a discrete cosine transform.
6. According to a preset compression ratio r, there are r*k*k values in the transformed matrix that will be sent to a higher layer and others will be replaced by zeros. The discrete cosine transform will be executed from the lowest layer to the highest layer recursively. When all compression values are obtained, the sink can recover all raw data through an inverse discrete cosine transform.

## 4. Simulation Results and Analysis

The basic theory and realization steps of the algorithm are given above. From the point of view of accuracy of recovered data, average energy cost of node and the number of expired nodes, in this section we make some comparisons and analysis through concrete simulation examples. Compared with the MSTC algorithm, the SC-LVLR algorithm has many merits, as follows:

1. Suboptimal clustering can gather a suitable amount of nodes which have spatial correlativity as a cluster, which is beneficial for eliminating spatial data redundancy, balancing the energy cost of networks and extending the longevity of the whole network. MSTC forms clusters evenly based on a virtual grid, which leads to some clusters including more nodes and the cluster heads expiring frequently and even some local holes may appear quickly.
2. The same compression dictionary is used to code the difference, both in eliminating spatial redundancy and time redundancy. The accuracy of recovered data can be regulated by changing the size of the coding dictionary. However, MSTC eliminates time redundancy by comparing differences with a threshold. If the threshold is too large, the accuracy of recovered data will be obviously affected, or time redundancy can’t be eliminated effectively.
3. Data spatial redundancy in the course of routing is eliminated based on a local virtual coordinate system, which is not only beneficial for reducing network congestion and improving the real-time ability of networks, but also beneficial for reducing the average energy cost of nodes, so the compressive performance of the whole network will obviously be improved. In MSTC, data from different adjacent nodes are mapped into a matrix, which is executed by discrete cosine transformation. For eliminating data spatial redundancy, a compression ratio is set and the transform coefficients are reduced correspondingly. However, when reducing transform coefficients directly according to a fixed compression ratio, this is not able to reflect any dynamic changes of the monitored data. If the ratio is too high, the accuracy of the recovered data will be greatly reduced, or the data spatial redundancy can’t be eliminated efficiently.

### 4.1. Algorithm performance evaluation model

To fairly compare SC-LVLR with MSTC, some efficient evaluation criteria should be used. Reducing average energy cost of nodes is one of the most important aims of designing data compression algorithms for wireless sensor networks, so the average energy cost of nodes is considered here as a performance evaluation criterion. Meanwhile, for measuring the accuracy of recovered data, signal to noise ratio (abbr. SNR) is also taken as one of the evaluation criteria. As one of the most straightforward ways to measure the longevity of networks, the number of expired nodes is considered as the third evaluation criterion of algorithm performance in this paper. The formula of energy cost of nodes in this paper refers to reference [12] and takes Strong ARM SA-1100A as an example. The total energy cost of nodes can be expressed as follows:

(28) $$E=E_{\mathrm{lp}}+E_{\mathrm{lt}}+E_{\mathrm{lr}}+E_{\mathrm{rt}}$$

In equation (28), *E_lp_* stands for the energy cost for computing in a node, and *E_lt_* stands for the energy cost for transmitting data between nodes in a surveillance area, and *E_lr_* stands for the energy cost for receiving data from a sink, and *E_rt_* stands for the energy cost for transmitting data to a sink. The formula for computing the energy cost for transmitting data is shown as follows [12]:

(29) $$E_{\mathrm{lt}}=E_{\mathrm{elec}}*k+{ɛ}_{\mathrm{amp}}*k*D^{2}$$

In the equation above *E_elec_* = 50*nJ/bit*, *ε_amp_ =* 100*pJ/b/m^2^*, *k* stands for the number of transmitted bits and *D* stands for the transmission distance. The calculation of *E_rt_* is similar to equation (29). The energy cost equation of receiving data is shown as follows [12]:

(30) $$E_{\mathrm{lr}}=E_{\mathrm{elec}}*k$$

The meaning of the parameters in equation (30) is the same as referred above [12]:

(31) $$E_{\mathrm{lp}}=\mathrm{NC}*{\mathrm{Vdd}}^{2}$$

In equation (31), the parameter *N* stands for the number of cycles per instruction for the main chip, and *C* stands for the average capacitance switched per cycle, Vdd represents voltage, and *C* approximates 0.67nf for Strong ARM SA-1100A.

For an ordinary processor, if the distance from node to sink is vastly larger than the distance from node to node, the energy cost of executing instructions can be ignored, so the formula of energy cost can be given as follows:

(32) $$E\approx E_{\mathrm{rt}}$$

Because cluster heads are responsible for communicating with all nodes within a cluster, the energy cost of cluster heads is larger than that of ordinary nodes. It is thus rational to take the average energy cost of all nodes in the sensor network as a performance evaluation criterion.

Peak signal to noise ratio:

(33) $$\mathrm{SNR}=10\text{log}[\frac{\mathrm{AM}}{\sum_{x=0}^{M-1}{\left(f(x)-\hat{f}(x)\right)}^{2}}]$$

Where *A* stands for the peak value transmitted by node, *M* stands for the number of group of input data, *f*(*x*) stands for input data, *f̂*(*x*) stands for output data.

### 4.2. Simulation experiment

**Figure 3.**
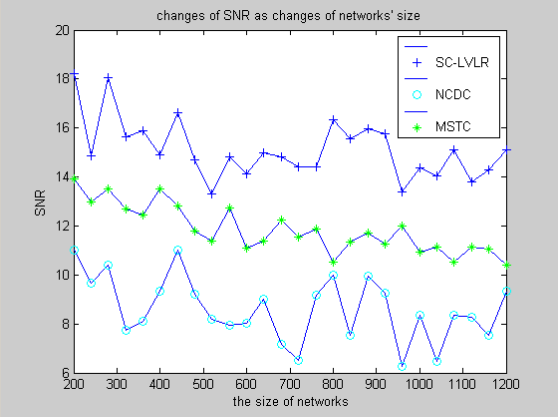
Changes of SNR as network size changes.

**Figure 4.**
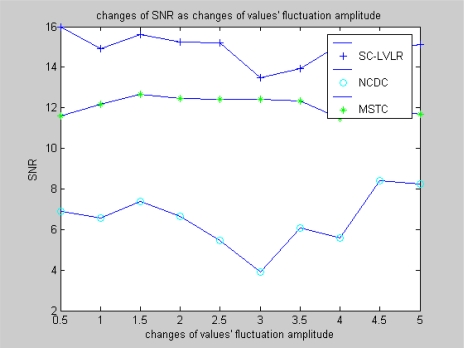
Changes of SNR with changes of values’ fluctuation amplitude.

Nodes are considered to be deployed randomly in the surveillance area. The following figures demonstrate comparisons of performance between SC-LVLR and MSTC. As a reference, the method of directly transmitting data from nodes to sink is included in the comparisons. Namely, there is no network clustering and data compression (abbr. NCDC) Operation. Both Figure 3 and Figure 4 are simulation comparisons of SNR. The difference is that the former aims to investigate the change of SNR with the change of sensor networks size under the condition that the change amplitude of monitoring value is 2, while the latter aims to investigate the change of SNR when the change of amplitude fluctuation of the monitoring value under the condition that the sensor network includes 1,000 nodes. From Figure 3, we can find that the SNR of SC-LVLR is higher than that of MSTC and NCDC as the networks size changes. The reason is that suboptimal clustering is used to gather nodes according to spatial correlativity in SC-LVLR, whereas a virtual grid is used to divide networks evenly in MSTC. Meanwhile, a discrete cosine transformation and preset compression ratio are used in MSTC, which will influence the accuracy of the recovered data and SNR correspondingly. Because a large amount of raw data needs to be transmitted from nodes to sink in NCDC, this not only leads to network congestion but also causes raw data losses, so the SNR of NCDC is the lowest in Figure 3. The situation of Figure 4 is similar to that in Figure 3.

**Figure 5.**
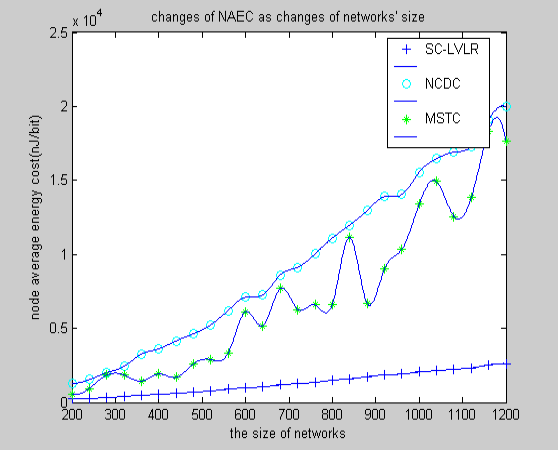
Changes of NAEC as network size changes.

**Figure 6.**
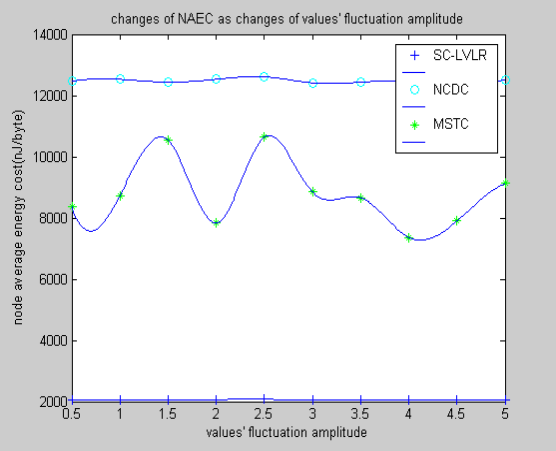
Changes of NAEC with changes of values’ fluctuation amplitude.

Figure 5 and Figure 6 reflect different algorithms’ performance of average energy cost of nodes in views of networks size and change of amplitude of monitoring values. From Figure 5, we can find that the average energy cost of nodes is increasing as the nodes increase. The reason is that the number of hops is increased as the nodes increase. The rate of increase of SC-LVLR is the lowest in the three modes. The fluctuation of energy of cost of MSTC is bigger than with SC-LVLR. The reason is that the fixed virtual grid leads the difference of energy cost of different cluster heads to be very large, so the practical deployment of nodes situation can obviously affect the average energy cost in MSTC. Meanwhile, for limited nodes resources, the discrete cosine transformation which is used for eliminating spatial redundancy in MSTC is too complex. However, difference coding which is used for eliminating temporal-spatial redundancy in SC-LVLR is simple and effective and the same coding dictionary can be used both for eliminating time redundancy and spatial redundancy, so the average energy cost of SC-LVLR is the lowest in the three modes discussed above. Under the condition that the sensor network includes 1,000 nodes, the performance of the three types of algorithm can be found in Figure 6 as the increase of change of amplitude of monitoring values. The reasoning is similar for Figure 5.

**Figure 7.**
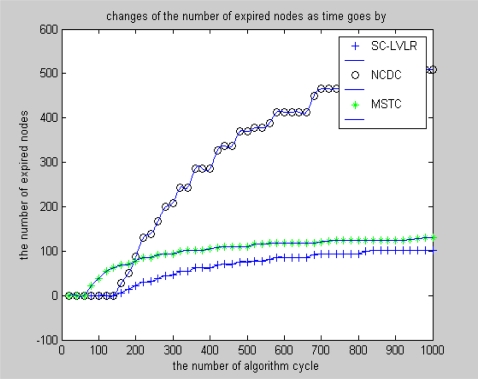
Changes of the number of expired nodes as time goes by.

**Figure 8.**
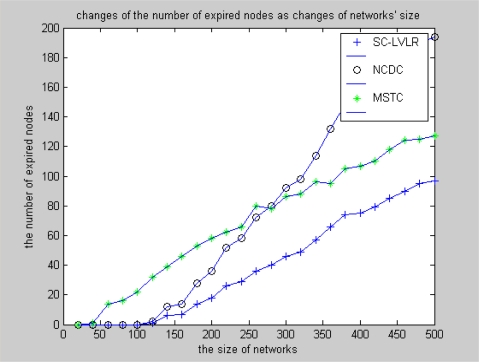
Changes of the number of expired nodes as network size changes.

**Figure 9.**
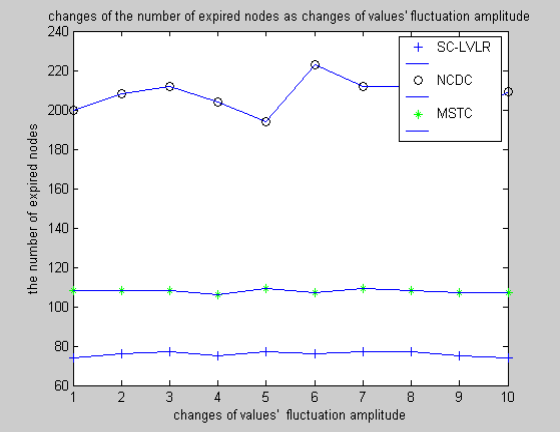
Changes of the number of expired nodes as changes of values’ fluctuation amplitude.

Figures 7, 8 and 9 reflect the performance of SC-LVLR, MSTC and NCDC in view of the number of expired nodes as the network working time, network size and change of amplitude of monitoring data change, respectively. Algorithm cycle is considered the measurement unit of network working time. From Figure 7, we can find that the number of expired nodes is increasing as time goes by and the increase rate of SC-LVLR is lower than for MSTC and NCDC under the condition that the network includes 1,000 nodes and the change of amplitude of monitoring values is 2. Specially, the number of expired nodes of MSTC is even larger than that of NCDC in the initial stage. The reason is that extra communication between nodes is needed in those virtual grids which include more than one node, and the cluster heads which cover these virtual grids will expire early, so holes caused by expired nodes and relative cluster heads may appear easier with MSTC than with SC-LVLR. Because there is no operation of clustering or alternating mechanism of cluster head, if holes appear in NCDC, hops will increase for transmitting formula amount of data and these holes will enlarge rapidly. The situation is demonstrated clearly in Figure 7.

Difference coding is used for eliminating time redundancy in SC-LVLR and the same way is used to eliminate spatial redundancy in courses of routing within and outside of clusters, so the actual data that must be transmitted is reduced considerably and the number of expired nodes is less in SC-LVLR than that in MSTC. Compared with difference coding along routing in SC-LVLR, more energy is needed in MSTC because the discrete cosine transformation is used in cluster heads to eliminate spatial redundancy. The simulation result is shown in Figure 8 under the condition that the network working time is 1,200 algorithm cycles and the change of amplitude of monitoring values is 2. The reason is similar to that in Figure 7. Under the condition that the network includes 1,000 nodes, changes of the number of expired nodes with the change of amplitude of monitoring values after 1,200 algorithm cycles is shown in Figure 9. From this figure, we can see that expired node fluctuations with MSTC and SC-LVLR are milder than that of NCDC. The reason is that there is no cluster head alternation mechanism in NCDC. Once a communication hole appears in a network, it will enlarge quickly because data routing have to bypass these holes and correspondingly more hops are needed.

## 5. Conclusions

With the features of limited energy, computation ability and memory of nodes and random deployment of nodes in sensor networks in mind, a data compression algorithm based on suboptimal clustering and local virtual coordinate routing has been proposed in this paper. Compared with MSTC, the relative complex discrete cosine transformation is avoided in SC-LVLR. After suboptimal clustering, routing within clusters can be obtained based on a local virtual coordinate system and an optimal global routing table can be obtained based on a global structure tree. Through coding difference of monitoring values in courses of routing within and outside of clusters, spatial redundancy can be eliminated near the place where spatial redundancy has occurred. Therefore, the amount of communication in networks can obviously be reduced and the average energy cost of nodes can be reduced and the longevity of the whole sensor network will be extended. The algorithm of this paper mainly aims to deal with one dimensional monitoring data. More redundancy may need to be eliminated when the monitored values are two-dimensional, for which the research on a corresponding algorithm is the key point of our next planned work.
